# Advancing sustainability in the food and nutrition system: a review of artificial intelligence applications

**DOI:** 10.3389/fnut.2023.1295241

**Published:** 2023-11-16

**Authors:** Zahra Namkhah, Seyedeh Fatemeh Fatemi, Amin Mansoori, Saeid Nosratabadi, Majid Ghayour-Mobarhan, Seyyed Reza Sobhani

**Affiliations:** ^1^Department of Nutrition, Faculty of Medicine, Mashhad University of Medical Sciences, Mashhad, Iran; ^2^Department of Biostatistics, School of Health, Mashhad University of Medical Sciences, Mashhad, Iran; ^3^International UNESCO Center for Health Related Basic Sciences and Human Nutrition, Mashhad University of Medical Sciences, Mashhad, Iran; ^4^Department of Nutrition, Electronic Health and Statistics Surveillance Research Center, Science and Research Branch, Islamic Azad University, Tehran, Iran

**Keywords:** food security, sustainable diet, nutrition science, artificial intelligence, machine learning

## Abstract

Promoting sustainability in food and nutrition systems is essential to address the various challenges and trade-offs within the current food system. This imperative is guided by key principles and actionable steps, including enhancing productivity and efficiency, reducing waste, adopting sustainable agricultural practices, improving economic growth and livelihoods, and enhancing resilience at various levels. However, in order to change the current food consumption patterns of the world and move toward sustainable diets, as well as increase productivity in the food production chain, it is necessary to employ the findings and achievements of other sciences. These include the use of artificial intelligence-based technologies. Presented here is a narrative review of possible applications of artificial intelligence in the food production chain that could increase productivity and sustainability. In this study, the most significant roles that artificial intelligence can play in enhancing the productivity and sustainability of the food and nutrition system have been examined in terms of production, processing, distribution, and food consumption. The research revealed that artificial intelligence, a branch of computer science that uses intelligent machines to perform tasks that require human intelligence, can significantly contribute to sustainable food security. Patterns of production, transportation, supply chain, marketing, and food-related applications can all benefit from artificial intelligence. As this review of successful experiences indicates, artificial intelligence, machine learning, and big data are a boon to the goal of sustainable food security as they enable us to achieve our goals more efficiently.

## 1. Introduction

The food system comprises production, processing, distribution and storage, food procurement, consumption, and waste (1). Food production and consumption will undergo significant changes in the next 30 years due to the growth of the global population by (8.5–10 billion in 2050) and other socio-economic developments (2, 3). Evidence shows that the food system can contribute significantly to climate change through excessive nitrogen and phosphorus inputs, using water sources, and greenhouse gas (GHG) emissions (3). GHG emissions are projected to increase by 87% from 2010 to 2050; also the demand for cropland use will rise by 67%, phosphorus application by 54%, and nitrogen application by 51% (3). Based on these changes, it is expected that the food systems’ environmental pressures will increase by about 50–90% in the absence of technological advancements and other mitigation measures (3). Thus, to achieve food security and sustainability, we must alter the current food system and consumption patterns.

Food security concerns and global warming have led to an interest in sustainable diets because of the solid environmental impact of diets (4). Therefore, to achieve food security, it is necessary to increase production sustainably. When considered in the context of sustainable food systems and a sustainable diet, the change in the food system may be feasible (5, 6). In the FAO’s definition, sustainable diets contribute to food security, nutrition, and health for present and future generations by being environmentally friendly. As well as being nutritionally adequate, safe, and healthy, a sustainable diet protects and respects biodiversity and ecosystems while optimizing natural and human resources (7).

Growing productivity at different stages of the production chain up to food consumption seems to contribute to the sustainability of the food and nutrition system, which ultimately leads to sustainable food security (8). Nevertheless, the question is, how can this be achieved? One solution could use artificial intelligence (AI). In its simplest form, AI is a technological branch of science that combines computer science with robust datasets to solve problems. AI initially was referred to as an ultimate goal and its related background science to address making machines as intelligent as humans are. Although helpful, this idea has evolved over the last decades. This is because not all our goals in building intelligent computation are to have them as smart as a human is, having all of a human’s limitations. In this regard, we need to identify our target application (9). AI allows identifying gaps across the food system to set a sustainable strategy (10). As well as the food system, AI is used in various fields, including medicine, healthcare, and nutrition. Therefore, AI can lead to positive outcomes, including monitoring the complete supply chain process, predicting disease risk, designing personalized nutrition decisions, and improving health (11–16). Herein, we review the current status of AI in the food system, nutrition, and health concerning improving the efficiency and sustainability of diets.

## 2. How AI relates to the food supply

AI, as a powerful tool for data analysis in food production, enables effective monitoring of the entire supply chain process. Machine learning (ML) and deep learning are widely used AI techniques (17). ML technologies are used in the preproduction, production, and processing phases and could also find applications in the distribution cluster, especially in storage, transportation, and analysis of consumer behavior (16). This utilization of AI technology not only facilitates more informed decision-making in the management of farm systems but also acts as a catalyst for the advancement of decision support and recommender systems (18). Following the content presented by Ahmed et al. (1), it is imperative to delineate the food system supply as a multifaceted entity encompassing several integral components. These components collectively make up the entire food system and involve production, processing, distribution, storage, food procurement, consumption, and waste management.

In the scope of our research study, we have elected to categorize and examine these components under three overarching sections: food production and processing, food distribution and consumption, and food waste management.

### 2.1. Food production and processing

The first stage of the agriculture supply chain is the preproduction phase, which pertains to crop yield, soil characteristics, and irrigation needs. ML algorithms use input data, such as equipment needs, nutrients, and fertilizers, to aid stakeholders and farmers in predicting crop yields and enhancing smart farming techniques (16).

Numerous studies have concluded that machine learning (ML) algorithms play a vital role in soil management techniques. These studies showed the effectiveness of ML methods in predicting critical soil parameters such as moisture content, organic carbon levels, and total nitrogen (19). Additionally, ML algorithms, when integrated into smart irrigation systems, have proven valuable for optimizing irrigation practices, enhancing crop quality and quantity, and effectively managing drought situations (20, 21).

Smart farms utilize an automated irrigation system that monitors and controls water tanks and open irrigation systems and chamber irrigation systems to optimize water resources (22). Using AI in crop management begins with the sowing of the crop and continues through the monitoring of its growth, harvesting, storage, and distribution. Several applications of AI are being used in farming, including crop health management, automation of farming operations (23), and demand-driven supply chains (24). Using AI helped resolve crop selection issues and improve net yields over the season (25).

In the agricultural supply chain, the production phase is crucial. Evaluating AI implementation in agriculture, including weather prediction, soil analysis, disease and pest control, crop quality management, and harvest optimization, is essential. There are many ML algorithm models for weather prediction (26), crop protection (27), weed detection (28), crop quality management (29), and harvesting (30).

Having a thorough comprehension of weather patterns aids in making informed decisions, leading to increased crop yields of superior quality (31). To efficiently manage and prevent diseases, farmers can implement an integrated disease control and management approach that comprises physical, chemical, and biological measures, with the help of AI technology (31, 32).

AI-powered systems and deep learning algorithms are utilized to analyze the information or data gathered by AI agents, facilitating the monitoring of crop and soil health (33, 34). For example, an artificial neural network (ANN) model predicts soil texture using hydrographic data derived from a digital elevation model (DEM), including sediment delivery ratio, terrain factor, and slope position (35).

AI and image processing have made significant strides in addressing the challenge of weed identification, as demonstrated in studies (36–38). These previous studies conclude AI models, particularly Support Vector Machines (SVM), are effective in determining optimal nitrogen application rates and excel at early stress identification during crop growth, highlighting SVM’s potential for enhancing crop yield with timely interventions (38). Additionally, one study underscores the economic significance of mitigating weed-related profit and yield reductions (39) and highlights the role of AI and ML in addressing spatial heterogeneity and its impact on crop yield (40, 41).

Research findings suggest that the use of AI-based technologies in agriculture and food production could have a positive impact on the environment (42–44). For instance, by 2030, these technologies might increase crop yields by up to 30%, save over 300 billion liters of water, and reduce annual oil consumption by 25 million barrels (45).

The third stage in the food supply chain is the processing cluster, encompassing a range of techniques, such as heating, cooling, milling, smoking, cooking, and drying for agricultural products. By employing a set of efficient parameters during this stage, it is possible to produce food products of high quality and quantity while minimizing resource usage.

Modern food processing technologies based on ML were supported by SVM and ANN models to identify the existence of nitrosamine in food samples of red meat (46). Additionally, AI-powered robots for harvesting cucumbers have been developed, featuring computer vision systems and hardware components like an autonomous vehicle, a manipulator, and an end-effector, which can detect and image the ripeness of cucumbers with a high level of accuracy (47). Furthermore, AI has showed its effectiveness in improving the drying process of fresh foods, fruits, and vegetables using physical fields like microwave, radio frequency, infrared radiation, and ultrasonic fields (48). For example, online detection and control of the drying process using AI helps reduce energy consumption, prevent uneven drying, improve sensory evaluation, and reduce nutrient loss (49).

### 2.2. Food distribution and consumption

In the distribution phase of the agriculture supply chain, the emphasis is on delivering safe, high-quality food to consumers. ML algorithms aid in tasks like inventory management, transportation, storage, and consumer analytics to minimize damage and uphold food quality (16). AI enables product tracing and safety assurance, optimizing supply chain management, and facilitating food safety testing and monitoring at every stage (50).

The importance of food safety cannot be overstated, and one way to achieve this is by using various methods, such as Image Processing (IP) to classify, identify, and recognize the quality of food products. IP systems utilize ultrasound, X-ray, near-infrared spectroscopy, and document scanners to analyze the size, shape, and texture of the product (51). This approach can also apply to product packaging to detect defects and grade quality (52, 53). In the realm of sustainable food systems, the application of AI has brought significant advancements, particularly in the areas of food grading and quality control within the food industry. Food grading, a crucial process for evaluating food product quality and safety, relies on predefined standards encompassing parameters such as size, shape, color, texture, flavor, freshness, and freedom from defects. AI technologies, encompassing computer vision, machine learning, and robotics, have helped to automate and enhancing this process. By enabling real-time inspection, classification, and sorting of food products, AI minimizes human errors, reduces labor costs, curbs wastage, and concurrently boosts productivity, profitability, and customer satisfaction (54–56).

Quality control, another indispensable facet of the food industry, revolves around the meticulous assurance that food products meet the exacting standards and safety requisites set by both customers and regulatory bodies. AI plays a pivotal role in elevating the precision and efficiency of this process. Leveraging machine learning, natural language processing, and predictive analytics, AI seamlessly aggregates, analyzes, and interprets voluminous data from diverse sources, including sensors, cameras, reports, feedback, and alerts. This data-driven approach proactively identifies potential issues and risks before they escalate, optimizes operational processes, bolsters transparency and traceability, and empowers data-informed decision-making (57, 58). The insights garnered from AI-driven analysis not only ensure optimal feeding and harvesting, but also underscore the critical role AI plays in enhancing the sustainability and efficiency of food systems (58).

The assessment of dietary intake relies heavily on nutritional data sourced from food composition tables or databases, which is a crucial aspect of evaluating the nutritional value of food. However, given the ever-expanding variety of food products and the rapid evolution of the food supply chain, traditional methods are struggling to keep pace in maintaining up-to-date food composition databases (59). As big data techniques are increasingly used by various fields in non-profits, science, business, and government to collect, store, process, and analyze data, this section introduces the AI approach to managing and evaluating food composition and food labels (60, 61).

Within the domain of sustainable food systems, the role of AI in food composition analysis and food labeling is paramount. Food composition analysis entails the intricate task of discerning the nutritional and chemical attributes of food products, encompassing crucial elements such as protein, fat, carbohydrates, vitamins, minerals, and antioxidants. AI is instrumental in automating and optimizing this process by employing innovative techniques like computer vision, spectroscopy, and machine learning to scrutinize food images or spectra, extracting pertinent information. This not only streamlines the process but also ensures the delivery of precise and dependable data that proves invaluable to consumers, producers, regulators, and researchers (44). In the broader context of sustainable food systems, AI’s contribution supports the development of healthier and more diversified dietary choices. For instance, an examination conducted by Liu et al. (62) underscored the potential of AI in food composition analysis, revealing its capacity to enhance the accuracy, efficiency, and resilience of these analytical methodologies, thus further reinforcing its pivotal role in this arena.

Continuing from the discussion of AI’s role in food composition analysis and its broader impact on sustainable food systems, it is noteworthy that exemplary projects like the Food Label Information Program (FLIP) 2020 highlight the practical application of artificial intelligence in developing comprehensive food composition databases. FLIP 2020, conducted in three phases between May 2020 and February 2021, focused on collecting data from Canadian food and beverage package labels offered by major e-grocery retailers. This data collection utilized Python-based website scraping and Optical Character Recognition (OCR) enhanced by AI. The project demonstrated the ability to autonomously collect data from online markets, leading to the development of a precise, transparent, detailed, and adaptable food composition database. This database is essential for monitoring the constantly changing food and beverage industry landscape (59). This practical example thus underlines the pivotal role AI plays in ensuring the integrity and accessibility of data crucial for the sustainability and transparency of food systems.

Another study explored the Michigan State University Environmental Science and Policy Program Annual Survey (2019) data to examine eight labels relating to food production techniques and customer preferences (63). Besides the statistical model, an ML analysis was conducted on raw input datasheets. This study utilized four ML predictive models, including logistic regression, SVM, random forest, and neural networks to prepare, verify, and test participants’ expected propensity to purchase or pay more for labeled products. According to this study, the label “raised without antibiotics” was associated with the highest average accuracy of the SVM learning model for predicting consumer willingness to buy. Furthermore, ML models offered an acceptable average prediction accuracy score for eight labels, introducing a new method for assessing data from food labeling surveys (63).

Over time, nutrition research has developed various classical dietary assessment methods; however, some challenges have remained (64). These methods are difficult to apply because they are paper-dependent, subjective, time-consuming, and prone to systematic errors (65, 66). Innovative dietary assessment tools based on AI that utilize different sensors, software, or image/voice-based approaches have improved health outcomes in line with technological advancements (11–15). As nutrition is among the healthcare fields that are increasingly benefiting from these new computational techniques, mainly because of the significant amount and complexity of data generated in nutrition research, these ML functions potentially apply. For example, researchers developed a sound-based recognition system that analyzed acoustic variables using an ear-pad gadget with a tiny microphone inside that measured the weights of bites of apple, mixed salad, and potato chips, with 94 percent accuracy (67). Speech2Health is another example of a voice-based mobile nutrition monitoring system that applies speech processing, natural language processing (NLP), and text mining techniques on a single platform to promote nutrition monitoring (68). The results of the experimental study indicated that Speech2Health had an accuracy of over 90% in computing calorie intake (68). In another study, Mertes et al. (69) presented a standalone plate system capable of measuring the weight and location of bites during unrestricted eating by utilizing a supervised learning method. This system correctly identified 602 bites out of 836 actual bites with a precision of 0.78 and a recall of 0.76.

Another illustrative example is the Snap-n-eat application, which utilizes an SVM classifier to recognize food items and assess nutrient and energy intake from photos taken on a mobile device (70). Users photograph their food, and the system isolates the relevant portion, discarding the background. A linear SVM classifier analyzes these sections, using features from various locations and scales to identify the food. This process culminates in portion size determination and estimation of the food’s caloric and nutritional content (70).

The eButton, a tiny computer with a camera integrated into a 6-centimeter button worn on the chest, was another significant image-based device (71). As the eButton is pinned in this location, it can access data from the external environment and the internal space of the body as it is very close to the heart and lungs. At a predetermined rate, the eButton snaps pictures, for example, one photo every two seconds while a meal is consumed. In theory, the images can be analyzed by an algorithm to determine the food item and portion size based on color, texture, plate shape, and eating utensils. Therefore, the calories and nutrients can be derived from a linked dietary database by providing information on the food item and serving size (71).

Advanced computing plays a vital role in clinical nutrition, complementing nutritional epidemiology. Clinical data in this field aid in predicting disease risk, conducting outcome-based research, and personalizing decisions. For example, researchers used a multivariable logistic regression model combined with machine learning to assess adverse pregnancy outcomes in over 7,500 pregnant women based on fruit and vegetable consumption. Surprisingly, the ML model revealed a reduced risk of preterm birth, small-for-gestational-age birth, and preeclampsia in those who consumed the most fruits and vegetables, contrary to expectations (72).

Berry et al. (73) aimed to predict the postprandial values of glucose and triglycerides (TG) in over 1,000 healthy adults in the United Kingdom using an ML model. Researchers used a random forest model to predict postprandial TG and glucose based on relevant information (such as meal composition to microbiome and biochemical parameters). The model predictions for TG and glucose rise were *r* = 0.42 and *r* = 0.75, respectively (73).

In a study investigating colorectal cancer prediction, researchers examined the interplay of diet, genetics, and related factors using the healthy eating index (HEI) on 53 colorectal cancer patients and 53 family/friend pairs (74). They employed various techniques, including data visualization, identifying familial dependencies, ensemble methods for variable importance, for predictive modeling. Shiao et al. (74) concluded that genetic polymorphisms in folate metabolism and dietary factors could predict colorectal cancer. This suggests individuals with such single nucleotide polymorphisms (SNPs) may consider dietary adjustments based on this study to mitigate disease risk (74).

### 2.3. Food waste management

Approximately 1,300 million tons of food are discarded annually. It is estimated that 25% of this food can feed the 795 million malnourished people around the world (75). Sustainable food production and consumption present formidable challenges across the food supply and distribution systems. Addressing these challenges is possible through the innovative integration of AI, offering a novel approach to curb food waste at various stages of the food cycle. AI emerges as the tool of choice in tackling food security issues, simplifying tasks from weather prediction to curbing food waste accumulation.

Khan et al. (76) introduced an innovative waste management system using ultrasonic and moisture sensors on trash bins. These sensors detect fill levels and moisture content, with data processed by an Arduino and uploaded to an online dataset. Drivers can access a mobile app to locate nearby trash containers and check their status (full/empty, wet/dry), optimizing garbage collection. This system enhances environmental friendliness by categorizing waste and predicting site-specific waste levels through image processing. Waste-collection vehicles prioritize their routes, saving time. However, implementation costs are a challenge for governments, though long-term cost-effectiveness is expected (76).

As an additional example, Amani and Sarkodie (77) introduced a deep learning-based method for detecting spoiled meat, a significant contributor to food waste and greenhouse gas emissions. Traditional meat supply chain management methods, prevalent in many developing countries, rely on manual monitoring and non-intelligent systems, which are prone to human error. Detecting spoiled meat in time is crucial to reducing waste and preventing the spread of bacteria. AI, powered by deep learning and image processing, rapidly identifies and separates rotten meat from fresh meat. By training on various meat images, these AI systems become adept at detecting spoilage (77).

Faezirad et al. (78) introduced a novel model utilizing ML to combat food waste, particularly in university settings in Tehran, Iran. The reservation system, used to estimate food demand, sometimes led to unexplained meal waste. To address this, a more stable demand prediction mechanism was essential. Their approach involved integrating an ML model with the reservation system, considering many factors impacting student demand: day of the week, consecutive holidays, food attributes (price and name), reservations categorized by academic degree and accommodation, and attendance history. Individual factors such as academic level, housing, and dining hall participation were also included. By applying this integrated model, food waste was reduced by a remarkable 79% (78).

## 3. Discussion

According to the results of our research, AI has had a significant impact on different phases of the agriculture supply chain, including food procurement, consumption, and waste management. As shown in Table 1, it plays a crucial role in improving crop quality, health, and yield forecasting, as well as optimizing agricultural product processing to avoid overutilization of resources. AI is highly developed for assessing food security, quality, and safety, aided by advancements in nanotechnology and biotechnology. In food labeling and procurement, AI helps build comprehensive food composition databases. Innovative AI-based dietary assessment tools improve health outcomes by computing calorie intake and portion sizes. AI-driven waste management technologies, like Ultrasonic sensors and ML, enhance garbage collection safety and detect meat spoilage. Overall, AI technologies are vital for enhancing the food supply chain, raising productivity, promoting health, and efficient waste management.

**Table 1:** Key AI applications for enhancing sustainability in the food and nutrition system.

How AI relates to sustainable food systems?
Improving crop health and biodiversity » reduce the use of chemical pesticides and fertilizers, enhance carbon sequestration, and protect wildlife habitats Automating farming operations and optimizing supply chains » reduce labor costs, food waste, greenhouse gas emissions, and transportation distances Developing alternative and innovative food products » reduce the environmental impact of animal agriculture, such as land use, water use, and methane emissions

Section	Main points
Food production and processing	In preproduction, AI aids in crop yield prediction, soil management optimization, and the advancement of intelligent irrigation systems. Within the production phase, AI encompasses crop health monitoring, farm operation automation, weather forecasting, disease and pest control, weed detection, crop quality management, and harvesting optimization. The implementation of AI in agriculture carries the potential for positive environmental impacts, including increased crop yields, efficient water resource utilization, and reduced fuel consumption.
Food distribution and consumption	AI is employed to ensure food safety, traceability, quality control, and effective inventory management. In the consumption phase, AI is utilized to analyze consumer preferences, behavior, and feedback, which helps businesses to improve their products and services. AI is deployed to collect and update food composition databases, using advanced techniques such as web scraping and optical character recognition. AI is utilized to assess the impact of food labeling on customer choices. The use of AI in the food industry has made a significant impact, providing businesses with an effective tool to manage inventory and enhance customer satisfaction.
Food assessment and clinical nutrition	AI is harnessed for dietary intake assessment via sensor technology, software applications, and image/voice-based methods. AI mitigates limitations associated with traditional dietary assessment techniques, such as reliance on paper-based records, subjectivity in evaluations, time-intensive processes, and potential systematic errors. AI contributes to disease risk prediction, outcome-driven research, and the customization of nutrition guidance through the utilization of clinical data. AI is instrumental in the analysis of post-meal glucose and triglyceride levels, employing a sophisticated random forest model.
Food waste management	AI is deployed in food waste reduction initiatives across different stages of the food cycle, using technologies such as sensors, image processing, deep learning, and ML techniques. AI contributes to the detection of spoiled meat, the optimization of waste collection routes, categorization of waste types, and the forecasting of food demand dynamics. AI has the potential to yield environmentally positive effects, including the reduction of greenhouse gas emissions, the preservation of limited landfill capacity, and the mitigation of food scarcity concerns.

Enhancing productivity within the food and nutrition system is an imperative step toward achieving sustainability on multiple fronts. The contemporary global food landscape faces formidable challenges, including a growing population, increased demand for diverse diets, resource limitations, and environmental concerns. In this context, the need to produce more food with fewer resources while minimizing negative impacts becomes clear (2, 4). Additionally, advancing sustainability in food and nutrition systems is crucial to addressing a variety of challenges and trade-offs within the existing food system. This imperative is underlined by several key principles and actionable steps: (1) Enhancing productivity, employment, and value in food systems through innovation and efficiency; (2) Reducing waste and adopting sustainable agricultural practices to safeguard natural resources; (3) Improving economic growth and livelihoods, including fair income and social protection; and (4) Enhancing individual, community, and ecosystem resilience, including disaster risk reduction and climate adaptation. By advancing sustainability, multiple benefits can be achieved, benefiting human health, the environment, and society. As a result of this approach, we can meet the nutritional needs of all people without compromising the planet’s health or the well-being of future generations (79–81). As discussed in the following section, AI technologies play a crucial role in achieving these sustainability principles and improving our capacity to address food system challenges and tradeoffs.

AI technology is a critical element of the food supply chain and is making its way into all aspects of the food system, offering the potential to create the sustainable food system we require. Considered a technological solution, AI is expected to enhance the efficiency and productivity of food and nutrition systems, playing a key role in achieving a sustainable food system (82). The pivotal role of AI in advancing food sustainability has been shown across four key domains: crop health and biodiversity, food insecurity, food waste, and food quality and safety (83). AI technologies are leveraged to promote sustainable farming practices, predict hunger, reduce food waste, and enhance food safety, offering significant benefits to human health, the environment, and society. For instance, through the use of machine learning, natural language processing, and computer vision, it’s possible to develop innovative food products that have less of an environmental impact. Specifically, plant-based or cell-based foods that mimic the taste, texture, and nutritional value of animal-based foods can help reduce the negative effects of animal agriculture, such as land use, water consumption, and methane emissions (84).

To effectively implement AI in food and nutrition systems, it is essential to take concrete actions. Governments and organizations should prioritize the development of pragmatic strategies for seamlessly integrating AI throughout the entire food supply chain, from preproduction to consumption. As AI technology continues its evolution, it has the potential to make significant contributions to the creation of sustainable diets. Given the dynamic nature of this technology, it is imperative to conduct ongoing research in this field and remain up-to-date with the latest advancements (85).

As this study emphasizes advances in AI and the food and nutrition system, it suggests that government officials, researchers, and companies are coming together at the global level to improve food systems’ efficiency and productivity (86). Consequently, the success of organizations will depend on their ability to innovate operations, products, and services by designing, implementing, and monitoring AI strategies (87).

Last but not least, the findings of this study confirm the importance of interdisciplinary studies between the sciences related to food and nutrition and other sciences, here computer sciences including AI technology. Integrating AI in the food and nutrition sector is an exciting prospect, but it also comes with its fair share of challenges. There are ethical, social, and practical concerns that need to be addressed to ensure that the integration is successful. For instance, we need to make sure that AI algorithms are free from bias and that the data collected is secure and private. There’s a need to ensure that human rights are respected, and that AI adoption does not widen the gap between the haves and have-nots. Lastly, we need to beef up cybersecurity to protect against cyberattacks. All these aspects require careful consideration and regulation to ensure that AI contributes positively to the food and nutrition sector (88, 89). Therefore, developing a sustainable food and nutrition system, and creating real transformation at a larger scale, requires connections and uniting voices while respecting and recognizing diversity. Bringing together academia, society, and industry in a transdisciplinary setting is crucial here, as these future challenges cannot be solved by one alone (90).

## Author contributions

ZN: Methodology, Project administration, Writing – original draft, Writing – review & editing. SF: Visualization, Writing – original draft, Writing – review & editing. AM: Writing – original draft. SN: Writing – original draft, Writing – review & editing. MG-M: Methodology, Writing – review & editing. SS: Methodology, Supervision, Visualization, Writing – original draft, Writing – review & editing.
